# SVANET: A Smart Vehicular *Ad Hoc* Network for Efficient Data Transmission with Wireless Sensors

**DOI:** 10.3390/s141222230

**Published:** 2014-11-25

**Authors:** Prasan Kumar Sahoo, Ming-Jer Chiang, Shih-Lin Wu

**Affiliations:** 1 Dept. of Computer Science and Information Engineering, Chang Gung University, Kwei-Shan 33302, Taiwan; E-Mail: pksahoo@mail.cgu.edu.tw; 2 Dept. of Electrical Engineering, Chang Gung University, Kwei-Shan 33302, Taiwan; E-Mail: d9621006@stmail.cgu.edu.tw

**Keywords:** VANET, WSN, IEEE 802.11p, IEEE 802.15.4, data transmission

## Abstract

Wireless sensors can sense any event, such as accidents, as well as icy roads, and can forward the rescue/warning messages through intermediate vehicles for any necessary help. In this paper, we propose a smart vehicular *ad hoc* network (SVANET) architecture that uses wireless sensors to detect events and vehicles to transmit the safety and non-safety messages efficiently by using different service channels and one control channel with different priorities. We have developed a data transmission protocol for the vehicles in the highway, in which data can be forwarded with the help of vehicles if they are connected with each other or data can be forwarded with the help of nearby wireless sensors. Our data transmission protocol is designed to increase the driving safety, to prevent accidents and to utilize channels efficiently by adjusting the control and service channel time intervals dynamically. Besides, our protocol can transmit information to vehicles in advance, so that drivers can decide an alternate route in case of traffic congestion. For various data sharing, we design a method that can select a few leader nodes among vehicles running along a highway to broadcast data efficiently. Simulation results show that our protocol can outperform the existing standard in terms of the end to end packet delivery ratio and latency.

## 1. Introduction

**Figure 1.**
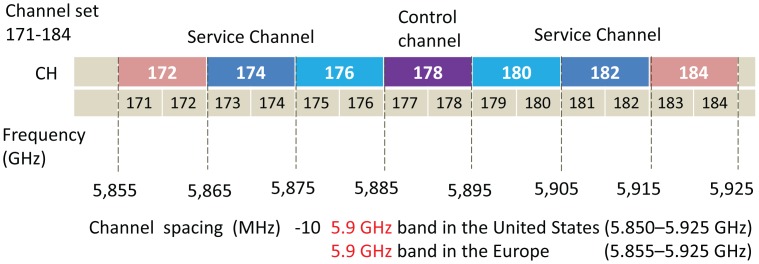
Spectrum allocation in IEEE 802.11p.

In 1999, dedicated short range communication (DSRC) has been allocated 75 MHz of the spectrum in the 5.9-GHz band for vehicle communications by the U.S. Federal Communication Commission (FCC). The DSRC spectrum used in the IEEE 802.11p standard [1] is divided into seven 10 MHz-wide channels, one of which is used as a control channel (CCH), and the others are used as the service channels (SCH). The CCH is used to transmit safety messages, and SCHs are used to transmit the non-safety messages, where vehicles need to switch between the CCH and SCHs to transmit messages. Normally, all safety messages, such as road accidents, are broadcast in the control channel and non-safety messages, such as audio, video and data, are exchanged in the service channels. However, the number of accidents on a highway may not be frequent, whereas downloading of data, audio and video is very common, as people prefer to download several services at a time. The channel time is divided into fixed time intervals, called synchronization (sync) intervals, as shown in Figure 1.

**Figure 2.**
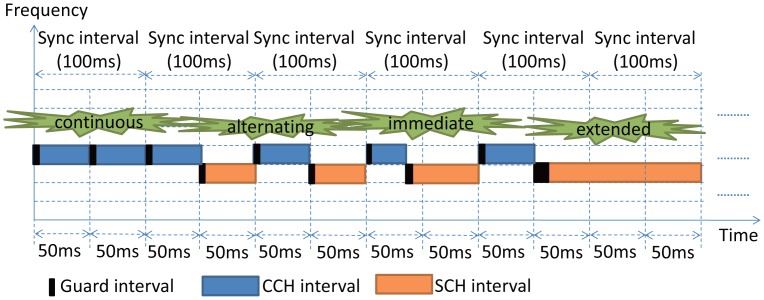
Standard channel access options in the IEEE 1609.4 standard.

According to the regulations in the IEEE 1609.4 standard [2], the channel time is divided into fixed time intervals, called synchronization (sync) intervals, and each sync interval has 100 ms, which is then equally divided into the control channel interval and the service channel interval of a 50-ms duration each, as shown in Figure 1. According to the standard, vehicles can transmit safety messages, HELLO messages and wireless access in vehicular environments (WAVE) service advertisements (WSAs) in CCH and non-safety messages in SCH. A vehicle transmits safety packets in the CCH interval. At the end of the CCH interval, if no non-safety messages are transmitted, it still continuously accesses the CCH. Otherwise, it switches to a SCH to transmit the non-safety message and goes back to CCH at the end of the SCH interval. Based on the standard regulations, there are four channel access options in the CCH and SCH interval: continuous, alternating, immediate and extended. As shown in Figure 2, a vehicle stays in the CCH to exchange safety messages in the continuous option or it can continue to stay in CCH if no service is available. In the alternating option, a vehicle accesses the CCH to transmit safety messages at the beginning of each CCH interval and switches to SCHs to transmit non-safety messages at the beginning of each SCH interval. In immediate SCH access situation, if a vehicle receives a request for this service, it will have immediate communication access to the SCH without waiting for the next SCH interval. The extended SCH access allows communication access to the SCH without any pause for CCH access. If a vehicle receives such a request, it shall begin to provide access to the indicated channel number unless a new request is received or it does not send any more requests for the extended access. After completion of the extended access period, a vehicle returns to continuous or alternating service channel access depending on which is applicable.

Based on the communication mechanism specified by vehicle *ad hoc* networks (VANET), data can be communicated among vehicles or between vehicles and roadside units (RSUs). Recently, on top of the VANET, applications have been developed to detect events, to collect and process the real-time information and to deliver them. A few researchers have proposed deploying road-side access points consisting of roadside wireless sensors, which are cost effective and allow one to create wireless sensor networks (WSNs), though they have energy and processing constraints. Those sensor nodes can use the IEEE 802.15.4 [3] medium access control protocol (MAC) to communicate with other sensors. The protocol operates on a direct sequence spread spectrum with the unlicensed 2.4 GHz band and can achieve a data rate of 250 kbps using O-QPSK modulation. At this frequency range, the band is divided into 16 non-overlapping channels. The medium access method of the MAC layer is the CSMA/CA protocol. In beacon mode, the standard can provide guaranteed transmissions through guaranteed time slots (GTS). It is to be noted that VANET does not provide any guaranteed real-time detection of road conditions or communication connectivity. It can be disconnected due to high mobility and unpredictable movements of the vehicles and the sparse deployment of RSUs. If the VANET is disconnected, any critical information about road conditions known by one disconnected subgroup of vehicles cannot be shared in a timely manner with other disconnected subgroups that need to know the information. Though deploying more RSUs seems to be a possible solution, it may significantly increase the investment cost. Wide area wireless networks (such as cellular networks) could be used to connect disconnected segments of the VANET. Though this approach may achieve communication connectivity, it does not solve the problem of guaranteed real-time sensing of road conditions. Wireless sensors, such as MicaZ motes [4], are much cheaper than RSUs. Besides, some inexpensive and low-power sensing modules, such as WiEye passive infrared sensors, have been commercialized and can be installed on the motes to sense the road conditions with a very low cost.

As the VANET cannot guarantee timely detection of dangerous road conditions or maintain communication connectivity when the network density is low, it may pose a big threat to driving safety. Besides, to detect single or multiple accidents of vehicles and then forwarding the corresponding messages in advance to other vehicles that are approaching the accident are highly important and essential. If an accident takes place on a highway, the most important activity is to provide a medical facility and other related help immediately. The time required for information flow from the accident to a nearby rescue team should be very critical. Hence, it is not only important to detect an accident in a timely manner, but it is also important to send the information about the location of the accident to the rescue team immediately. When sensors with GPS in each vehicle can detect the accident location, they can confirm that the accident has taken place, and the GPS can find the geographical location of the accident. It is to be noted that both VANETs and WSNs have lots of applications in common and are the subject of ongoing research activities, though the characteristics of VANETs and WSNs are very different. For example, nodes in WSNs are smaller in size, very resource and energy constrained and mostly static with respect to their position. However, they have good sensing capabilities to detect different types of events. In contrast, VANETs have very dynamic topologies and vehicles do not have energy constraints, though they cannot detect the events reliably. Vehicles could be equipped with sensors themselves to detect events. However, the sensor coverage of vehicles cannot be guaranteed, as vehicles are not present everywhere, and the data need to be transmitted from one location to another.

From the study of the existing literature on VANETs and WSNs, we are of the mind that the utilization of control and service channels in the current form is not efficient and needs to be adjusted dynamically. Moreover, an efficient data transmission method can be adopted by using inexpensive wireless sensors instead of deploying a greater number of RSUs. Accordingly, the wireless sensors can detect events, such as icy roads, accident and road traffic conditions and can disseminate messages through the on-board units (OBU) of vehicles by using the control channel. Since, transmissions of emergency messages due to event detections are not more frequent, vehicles on the highway can share audio, video, games and other multimedia content with passengers of other vehicles frequently. Accordingly, the transmitted data in our protocol can be classified into three categories based on their priorities. The rest of the paper is organized as follows. Related work is presented in Section 2. A hybrid smart vehicular network architecture is described in Section 3. Our proposed data transmission protocol is described in Section 4. A leader selection algorithm for sharing different types of data among vehicles is presented in Section 5. Performance evaluations of our work are given in Section 6, and conclusions are made in Section 7.

## 2. Related Work

The performance of the IEEE 802.11pprotocol is extensively studied in [5], and the authors show that this protocol has some problems, like transmission fairness, predictability, low throughput and a high collision rate, particularly in high-density networks. As analyzed in [6,7], vehicles moving with high speed can influence the throughput and reliability of VANETs. IEEE 1609.4 [2], a multi-channel extension of the IEEE 802.11p [1] standard, has been proposed to improve the service differentiation capability of the IEEE 802.11p standard. Much research has focused on application-based multi-channel issues for VANETs. In [8], the proposed multi-channel MAC for VANETs not only ensures the reliability of safety message transmissions, but also provides high throughput for non-safety data transmissions. In [9], the authors utilize on-board units inside the vehicles, as well as road side units (RSU) and suggest that vehicles must stay in CCH during the CCH interval to guarantee the reception of safety and control messages, while they may tune to the SCHs to transfer non-safety data.

The parallel usage of nearby channels incurs signal interferences among these channels. This kind of interference is known as adjacent channel interference (ACI) and has strong impacts on the efficiency of transmissions. In [10], the authors elaborate on the parallel usage of the CCH and SCH with adjacent channels and evaluate the effects on adjacent channel interferences. In [11], the authors suggest that the design of vehicular networks lies in the design of an efficient MAC protocol, which can be adaptable to different traffic scenarios. In [12], the authors extend the existing MAC protocols, which can dynamically adjust the length of CCH and SCH intervals according to the vehicle density and loading conditions of the network. The authors think that the dynamic adjustment of CCH/SCH duration in sync intervals based on the network density could make the use of VANETs more efficient and reliable.

In [13], the authors specify providing dynamic adjustment of the CCH interval duration for safety applications under various traffic conditions. In [14–16], the authors propose a variable duration of CCH interval (VCI) multi-channel MAC schemes, which can dynamically adjust the duration ratio between CCH and SCHs, as compared to the previous and current durations of CCH. They also calculate the optimum CCH intervals based on the number of vehicles and the length of messages. Much research has paid attention to cluster-based multi-channel MAC schemes in VANETs. In [17], the authors consider the reliability and connectivity of typical cluster-based protocol for VANETs on multi-channel scheme with power control to decide the cluster size and communication range. In [18], the authors also consider the power control and utilize the fuzzy-logic inference model as an adaptive learning mechanism to decide the cluster size and communication range. In [19], the authors adopt power control to reduce the transmission range, which could reduce the high overlap in high-density traffic and increase the transmission range to keep the connection seamless in low-density traffic.

Many researchers, such as [20,21], have proposed cluster-based multi-channel MAC protocols to improve the performance and reliability of VANETs. A clustering scheme is proposed in [22], where cluster heads have the main role of providing a TDMA schedule to their members. In [23], the authors proposed a clustering-based MAC multi-channel protocol (CMCP), where each node is equipped with two transceivers, and both transceivers can operate on different channels simultaneously. A mobility-based clustering scheme is proposed in [24]. The selection criterion for the cluster head depends on the Doppler shifts arising from received hello packets. The authors in [21] have proposed a mobility-based clustering scheme, which is called APROVE, using an affinity propagation algorithm to select cluster heads.

In [25], the authors propose a pre-crash detection system, which is equipped with a combination of different sensors to avoid obstacles, if there are any. In [26], the authors provide a reliable and timely dissemination of the detected dangers from the roadside sensors to vehicles that will be passing, in which sensors are deployed along the road to monitor and gather the information of weather and traffic conditions on a road segment for dangerous event detection. In [27], the authors designed distributed traffic information systems for detecting traffic flow problems on a road. In [28], the authors address different matters, including event detections and classifications, as well as the selection of appropriate communication protocols and some important design parameters. In [29], the authors evaluate the existing methods for vehicle event detection systems with video recorders. However, these systems incur many disadvantages, such as the inconvenience of deployment. In [30], the authors propose a cluster-based time-division channel allocation scheme. However, the service channel resources are wasted during the CCH time interval.

From the survey of the existing research, it seems that the most researchers have combined WSNs with VANETs. For this, many sensors need to be deployed, though we need only event-driven protocols for detection. Thus, we propose a cluster-based protocol to assist data transmissions, in which CCH and SCH intervals can be adjusted dynamically. The contributions of our work are summarized as follows.

In our work, a smart vehicular *ad hoc* network architecture is designed. In the architecture, there are a few sensors fitted into each vehicle, which is a very common way in most of state-of-the-art vehicles. The main contributions of our work are summarized as follows.

- We propose a hybrid vehicular *ad hoc* network taking wireless sensors and vehicles into account. Unlike other hybrid VANETs with sensors, sensors in our protocol are mobile, as they are fitted inside vehicles.
- In order to improve the speed and reliability of safety message transmissions, we use several road side sinks (RSSs) and a few smart road side units (SRSUs). In our protocol, SRSUs are present only near the interchanges of roads.
- In order to meet the demand of drivers and passengers to obtain various types of data, such as videos, audio, games and other related messages, we propose a priority-based data classification, in which different vehicles can access different data types, even if they are located within the same communication range.
- In order to avoid message redundancy in a dense VANET, we design weight-based leader selection algorithms to meet the users demand for different types of data.
- Though road accidents are normal, they may not take place frequently. Hence, we propose a dynamic utilization of control and service channels by adjusting sync intervals that supports safety and non-safety real-time and non-real-time data transmissions.

## 3. SVANET Architecture

In this section, we propose a hybrid architecture taking WSN and VANET into account, which is referred to as a smart vehicular *ad hoc* network (SVANET) throughout our paper. Besides, we categorize the types of data based on the priorities and assign service channels to the vehicles, so that different vehicles can access different data types, even if they are located within the same communication range.

### 3.1. The Hybrid Architecture

In our proposed architecture, there are SRSUs, which can communicate with the vehicles using the IEEE 802.11p channel access protocol, as well as with wireless sensors using the IEEE 802.15.4 MAC protocol. Since we consider the highway scenario, it is assumed that there are several interchanges to exit from the highway in case any event is reported. As mentioned earlier, the event could be an icy road, traffic congestion due to an accident or the presence of wild animals. An SRSU is supposed to wirelessly upload and download data from and to vehicles using either IEEE 802.11p or the IEEE 802.15.4 MAC protocol whenever any vehicle is passing within its communication range. It is assumed that there is one SRSU at least 2 km before each interchange, so that the SRSU can inform vehicles about any event by using the control channel, and drivers can make a decision to change their route in advance. In our proposed architecture, one ZigBee-enabled wireless sensor is fitted at the front end of each vehicle to detect events, which is very common nowadays in most modern vehicles. These wireless sensors operate in the unlicensed ISM 2.4 GHz band and use the IEEE 802.15.4 protocol for communicating with each other. In between any two SRSUs, there are several RSSs, which can receive data from wireless sensors using the IEEE 802.5.4 communication protocol. Each wireless sensor has the ability to sense and detect events. The RSSs are the sinks to collect data from sensors and can act as intermediate nodes to transmit the event information to the next hop RSS, which can ultimately be transmitted to SRSUs.

**Figure 3.**
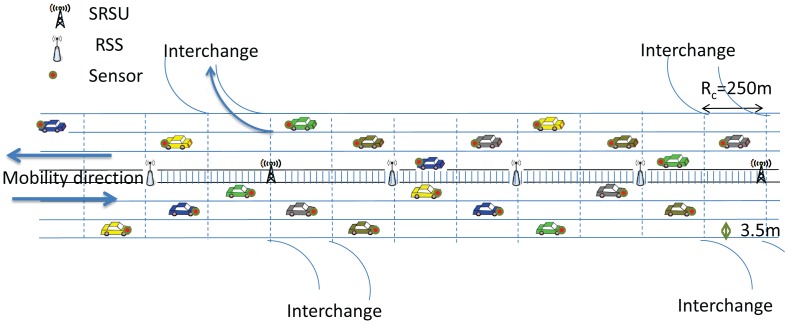
The proposed hybrid smart vehicular *ad hoc* network (SVANET) architecture.

Other than wireless sensors, a smart on-board unit (SOBU) is also present in each vehicle, which can act like a smart gateway to receive data from sensors for further processing. The SOBU can analyze the received data and then broadcast to other vehicle SOBUs within its communication range. It is to be noted that sensors fitted in each vehicle can transmit data to the SOBU of the same vehicle by using wired cables in order to avoid collisions or interference by using the wireless medium. Each SOBU communicates with other SOBUs wirelessly using the IEEE 802.11p protocol. In between any two SRSUs, the data are broadcast from one SOBU to another in a multi-hop fashion. As soon as a vehicle arrives within the communication range of any SRSU, the information of events from the vehicle's SOBU is uploaded to the SRSU using the IEEE 802.11p communication protocol, and the information of events received either from other vehicles or from the intermediate RSS is downloaded to the vehicle's SOBU using the IEEE 802.11p communication protocol. In the proposed architecture, sensors can communicate with other sensors or RSSs using the IEEE 802.15.4 communication protocol. Each RSS communicates with other ones using the IEEE 802.15.4 communication protocol. It is assumed that IEEE 802.11p and IEEE 802.15.4 coexist in each SRSU, and therefore, each RSS can transmit data to the SRSU using the IEEE 802.15.4 communication protocol. In order to make the architecture economically feasible, no cable or wireless connection is required in between any two SRSUs, as they can receive event information from vehicles, as well as from the RSSs to improve the data reliability. Any SOBU can communicate with other SOBUs using the IEEE 802.11p communication protocol. The proposed architecture for the highway scenario is shown in Figure 3.

### 3.2. Priority-Based Data Classification

In a highway, normally, people have to drive a long way for several hours and want to share various types of data, such as audio, videos, games and various offers from nearby gas stations. A VANET is the best way to transmit data from one vehicle to other, so that people in the vehicles can share the data and information anytime. Other than sharing data with each other, drivers need to know about the road and traffic conditions ahead of time, so that they can change their driving route in order to avoid traffic congestion. If an accident occurs on the highway, the timely information transmission from the place of the accident to a nearby rescue team for transporting emergency medical facilities is highly essential. Hence, such emergency information should be transmitted in a dedicated channel with the highest priority. According to the IEEE 802.11p standard, any emergency data can be transmitted in CCH. However, there are another six SCHs to transmit the data. In our protocol, the data that are shared with different vehicles can be classified into four different categories with different priorities, as shown in Table 1.

The safety message that is exchanged only in CCH is considered to be of the highest priority. The non-safety real-time data are considered to be of medium priority, and non-safety non-real-time data are considered to be of the lowest priority. Thus, all seven channels are divided on a priority basis. CCH is totally meant for exchanging the safety messages as emergency data and is therefore dedicated for the data with the highest priority. This safety message falls under the urgent category with Priority 1. Videos and graphics are considered to be Category I with Priority 2. Audio, images, commercial advertisements and non-real-time data are considered to be Category II and given Priority 3. Similarly, other non-real-time data, such as text messages and emails, are considered to be Category III with the lowest Priority 4. The data with Priority 1 is transmitted only in CCH, whereas the rest of the six SCHs are used to transmit the data of other categories. In the next subsequent sections, we design protocols to transmit data of different categories requested by different vehicles within the same communication range. As shown in Table 1, Category I data are transmitted either in Service Channels 1 and 2 (SCH1 and SCH2), Category II data are transmitted in Service Channels 3 and 4 (SCH3 and SCH4) and Category III data are transmitted in Service Channels 5 and 6 (SCH5 and SCH6).

Though road accidents are normal, they may not occur frequently, and therefore, CCH used for broadcasting the safety message cannot be used efficiently. Hence, the inefficient utilization of CCH may cause channel wastage due to fixed duration intervals between CCH and SCHs regulated in the IEEE 1609.4 standard. Due to the fixed duration of the channel interval, transmissions of safety and non-safety data cannot be handled efficiently. Therefore, in our data transmission protocol, it is proposed that the duration of CCH be reduced in the absence of safety messages and be used for the purpose of broadcasting the non-safety real-time and non-real-time data. Thus, the durations of CCH and SCHs can be adjusted dynamically to improve the channel utilization, which will be discussed in Section 5.

### 3.3. Data Collection and Forwarding by SRSUs

**Figure 4.**
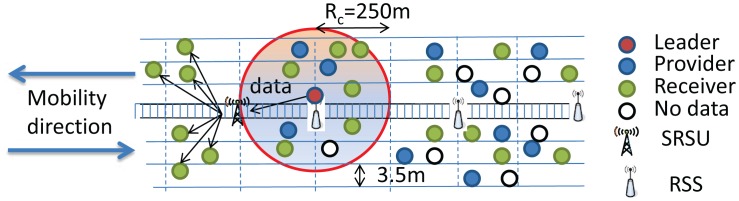
Data uploading to and downloading from the smart road side unit (SRSU).

In our protocol, the communication range of each vehicle (*R_c_*) is considered to be 250 m, and vehicles within the same communication range are categorized into four types. Vehicles having no data are termed as Null vehicles. Some vehicles in the VANET may have data of Category I, II or III, which they can share with other vehicles. Vehicles having data to share with others are termed as Providers, while vehicles getting data from others are termed as Receivers. Besides, one provider among all providers of each category of data is selected as a Leader, which will be discussed in Section 5. The role of leaders is to forward data to the next hop receivers. In our protocol, every vehicle has to upload safety messages whenever it enters the communication range of an SRSU, as shown in Figure 4. Similarly, the SRSU downloads messages to vehicles moving along the upstream and to the next hop RSSs for forwarding messages to the next hop SRSU. It is to be noted that communications between SRSUs and vehicles are made by using the IEEE 802.11p communication protocol, whereas communications between SRSUs and RSSs are made by using the IEEE 802.15.4 communication protocol. Once the SRSU collects messages, it broadcasts the messages to vehicles running along the opposite direction of the highway so that messages can be propagated very fast. The detail of the data transmission procedure is described in Section 4.

The format of each data packet is represented as ${\text{Data}}_{\#\text{Data},\#\text{node}}^{\text{side}}$, where the *side* represents the mobility direction of a vehicle, which could be upstream (*u*) or downstream (*d*). The symbol #*data* represents the data sequence number, which is used to fuse data and to maintain data integrity. The symbol #*node* represents the ID of a vehicle, which is a unique number, such as the license plate number of a vehicle. The #*node* is used to avoid data redundancy. For example, $D_{1,1}^{u}$, $D_{1,2}^{u}$, $D_{1,3}^{u}$, $D_{1,4}^{u}$, $D_{1,5}^{u}$, …, $D_{1,m}^{u}$ can represent the data received by an SRSU from the vehicles moving along the upstream direction and $D_{2,7}^{d}$, $D_{2,9}^{d}$, $D_{3,10}^{d}$, $D_{3,8}^{d}$, $D_{3,6}^{d}$, …, $D_{4,k}^{d}$ can represent the data received by the SRSU from the vehicles moving along the downstream direction. Here, $D_{1,3}^{u}$ implies that data with Sequence Number 1 have come from the vehicle with ID 3, which is moving along the upstream (left-hand side of the road). Similarly, $D_{3,2}^{d}$ implies that data with Sequence Number 3 have come from the vehicle with ID 2, which is moving along the downstream (right-hand side of the road).

Upon receiving data from the vehicles, the SRSU analyzes those data. If the content of the data is the same, the SRSU drops it; otherwise, it keeps the data for forwarding to other vehicles. Each vehicle that enters the communication range of the SRSU has to download the data and carries it for forwarding. Normally, the SRSU will transmit the data coming from downstream vehicles to upstream vehicles and *vice versa*. In our protocol, each vehicle has to download data using Service Channels 5 and 6, as they are assigned the lowest priorities.

## 4. Data Transmission Protocol in SVANET

It is assumed that each vehicle has one antenna and a GPS device, which is very common in most modern vehicles. As mentioned early, any vehicle that has data to send is termed as a Provider, and others are termed as Receivers. The transmission protocol is designed for the SVANET for a highway scenario with *m* lanes along each side. SRSUs are placed only before interchanges, and any two SRSUs are separated for *k* km (the value can vary depending on the distance between interchanges). Any sensor node in a vehicle senses events, aggregates the measured values and transmits to the SOBU of the vehicle. Simultaneously, the SOBU broadcasts the same information to nearby vehicles and RSSs within its communication range. The information sent by the sensor to the SOBU could include various physical quantities, such as temperature, humidity, surface conditions on the roads and also detected moving obstacles (e.g., animals). The received information is processed in the SOBU and potentially displayed to the driver of the vehicle. Once the processed sensor data is interpreted as a dangerous situation, the SOBU triggers a safety warning message immediately. The generated warning message is distributed by the SOBU to all vehicles in a certain geographical region, potentially using wireless multi-hop communications. The duration of the safety warning message is considered to be 100 units, which is partly used for CCH and is partly used for SCH. The backoff time is set in such a way that a vehicle that is far away from the location of the accident (event) can broadcast first for the acceleration of the message propagation. As a result, vehicles that receive the information are warned about dangerous spots in advance and can perform some appropriate countermeasures. It is to be noted that in our protocol, the propagation of an identical message is limited within two consecutive SRSUs. As soon as any vehicle carrying the message comes within communication range of an SRSU, it has to upload the message to the SRSU. Additionally, it also needs to download any new messages cached in the SRSU, which it carries until reaching another SRSU.

If a car accident happens on the road without any warning message, any moving vehicle close to the site of the accident may be in an emergency situation. Drivers typically decide their own braking actions depending on the tail brake light of the car immediately ahead. The duration between when an event is observed and when the brake is actually applied is considered as the drivers' reaction time, which typically ranges from 0.75 to 1.5 s [31]. Because of the tiny reaction time, vehicles moving at a high speed may not have enough time to stop and cause an overtaking collision, and the situations may be even worse. The other vehicles close to the site will slow down their speed and may cause a serious traffic jam until the accident is solved. Instead of a periodic transmission, we propose a triggered scheme in our protocol. Once a new vehicle is present, data should be transmitted, and therefore, the vehicle presence can be regarded as a special type of event detected by special sensors. Once triggered, the actual transmission from sensors to vehicles is a time-crucial task. As sensor nodes have a limited transmission range, the data need to be sent within a very short time. Vehicles usually move with relative speeds up to 30 m/s on highways, and as a delay between vehicle detection and the message transmission, the time frame left may be lower than a second. Hence, all messages being transmitted must be compacted into one or very few frames in order to improve the probability of successful transmission. It is suggested to transmit the event information faster to let every vehicle on the road know this information to avoid the site of the accident to prevent an overtaking collision. In the following subsections, we discuss how to transmit the message in each sync interval in a very efficient way.

### 4.1. Initial Phase

In the initial phase, vehicles have to sense the control channel first. If a vehicle senses the channel as idle, it broadcasts a HELLO message to other vehicles that are within its communication range. The HELLO message contains the ID, current velocity and location of the vehicle. However, if the channel is sensed as busy, the vehicle will wait for a random backoff time to avoid the collision with another potential transmission. At the end of each backoff time, the vehicle broadcasts the HELLO message. Upon receiving HELLO messages, each vehicle knows the ID, velocity and location of its one-hop neighbors.

### 4.2. Single-Hop Event Broadcasting Phase

**Figure 5.**
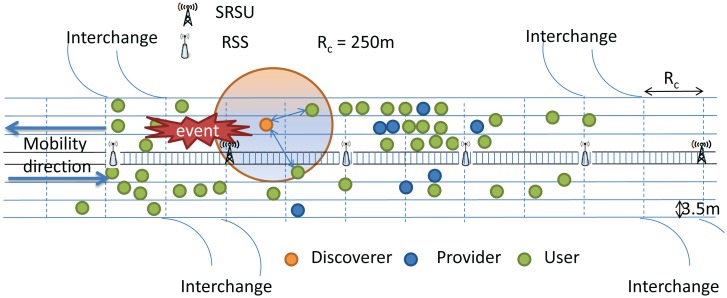
Example of event broadcasting phase.

In this phase, if a vehicle discovers the event related to a warning message, which can be the lane change information or an accident in the highway, it broadcasts the warning message in the CCH intervals, as shown in Figure 5. Other than the information about the event, the message also includes the Discoverer's ID, location (*x_D_*, *y_D_*), speed, moving direction, event ID, event location and time stamp of the event. Since, a vehicle may receive more than one warning message, a discoverer'sone-hop neighbors may ignore the repeated message after verifying the location and event ID of the event and then rebroadcasts the message to its next hop neighbors. A receiving node (User) goes to rebroadcast the backoff before forwarding the message to its next hop neighbors. Assuming the location of the User node is (*x_self_*, *y_self_*), the rebroadcast backoff time can be calculated as given in Equation (1). We use the projection distance instead of the distance between any two users, as we think the rebroadcast user is not only away from the discoverer, but also close to the next hop SRSU. The direction vector in Equation (1) can be easily calculated by (*x_SRBU_*, *y_SRBU_*) − (*x_D_*, *y_D_*). During the backoff procedure, if a vehicle receives the same warning message rebroadcasted from its one-hop neighbors, the vehicle abruptly stops the backoff procedure and drops the warning message. Otherwise, it rebroadcasts the warning message after the backoff time. Besides, if it receives the event ID from another vehicle that is behind it, the vehicle will ignore the message.

(1) $$\frac{1}{\text{Projection distance}}=\frac{\left|\text{direction vector}\right|}{\left|{\left(\text{direction vector}\right)}^{\cdot }\left(x_{\text{self}}-x_{D},y_{\text{self}}-y_{D}\right)\right|}$$

### 4.3. Multi-Hop Event Broadcasting Phase

Discovers—vehicles detecting an event related to a warning message—need to send the message to the SRSU that is located at the previous interchange of the event location. For example, the SRSU will be the rightmost SRSU in Figure 5. This is because we want to warn the incoming vehicles, which have the choice to get off the highway through the interchange to avoid traffic congestion.

**Figure 6.**
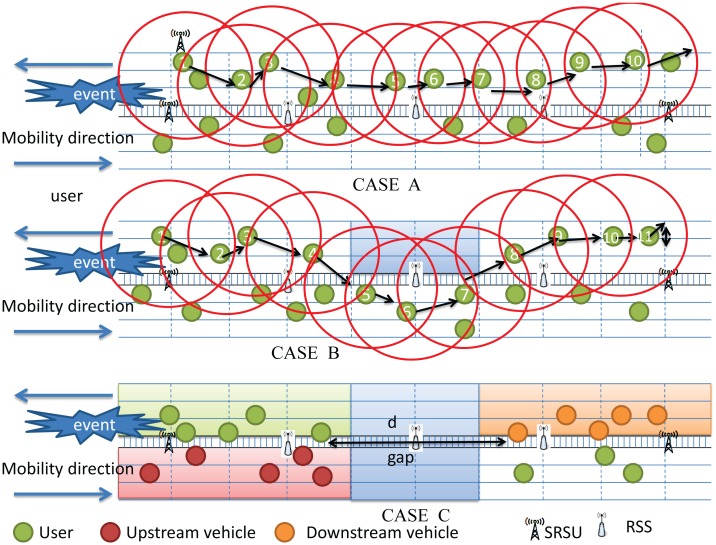
All possible ways of message forwarding. (**i**) Case A: message forwarding through vehicles moving along the same direction (downstream); (**ii**) Case B: message forwarding through vehicles moving along the opposite direction (upstream); (**iii**) Case C: message forwarding through RSS when the network is partitioned.

There are several different ways of message forwarding from the discover to the SRSU, as shown in Figure 6. It could be possible that the message is forwarded through the vehicles moving along the same direction irrespective of their mobility in the same or different lanes. Here, the intermediate vehicles between them can form a connecting path to forward the message, as shown in Case A of Figure 6. Assume that the message forwarding through intermediate vehicles to the SRSU requires *s* hops. Each message broadcast time can be calculated in Equation (2).

(2) $$\text{Broadcast Time}=\sum_{i=1}^{s}{\text{Broadcast Time}}_{i}$$

In another scenario, the condition is the same as Case A, except that at least two subgroups of vehicles cannot communicate with each other along the same moving direction, as shown in Case B of Figure 6. Therefore, a connecting path is not possible by the vehicles moving along the same direction. They can only form two connecting subpaths instead. In this case, a connecting subpath is established by vehicles moving along the opposite direction in addition to the same direction. Finally, a full connecting path can be formed by combining the three subpaths. Assume that the message forwarding by the vehicles moving along the same and the opposite direction are of *s* and *o* hops, respectively. The rebroadcast backoff time and broadcast time can be calculated in Equations (3) and (4), respectively, where (*x_p_*, *y_p_*) is the location coordinate of the message provider (last broadcaster) and *α* is the direction factor.

(3) $$\text{Reboardcast backof f time}=\frac{1}{\text{Projection Distance}}+\alpha =\frac{\left|\text{direction vector}\right|}{\left|{\left(\text{direction vector}\right)}^{\cdot }\left(x_{\text{self}}-x_{p},y_{\text{self}}-y_{p}\right)\right|}+\alpha ,$$

(4) $$\begin{array}{c} \text{Broadcast Time}=\overset{s}{\underset{i=1}{\text{∑}}}{\text{Broadcast Time}}_{i}\\ +\overset{o}{\underset{j=1}{\text{∑}}}{\text{Broadcast Time}}_{j}=\overset{s+o}{\underset{k=1}{\text{∑}}}{\text{Broadcast Time}}_{k}+\alpha *o \end{array}$$

It could be possible that there is a communication gap existing between two subgroups of vehicles in the forwarding path. In other words, the forwarding path to the SRSU is broken no matter which direction the vehicles are moving. Hence, the network is fully partitioned, as shown in Case C of Figure 6. Right now, there are three groups of vehicles. Let Group *I* be the set of vehicles that carries the event information, Group *J* be the set of vehicles that has no information about the event, and Group *K* be the set of vehicles that moves along the opposite direction and has knowledge about the event. Let *I_i_* be the vehicles in front of the gap, *J_j_* be the vehicles behind the gap and *K_k_* be the vehicles moving along the opposite direction. Let *d_a_*_,_*_b_* be the distance between any two vehicles *a* and *b* in general and *V_i_*, *V_j_*, *V_k_* be the velocities of the vehicles *i*, *j* and *k*, respectively. Now, the average waiting time for the vehicles moving along the same direction can be calculated by Equation (5).

(5) ∑i=1I∑j=1JdistJj,Ii−RcυIi−υJjI*J

The average waiting time for the vehicles moving along the opposite direction can be calculated by Equation (6). Finally, the message can be carried out by the vehicles moving along the opposite direction and is forwarded to the next SRSU through several hops. In addition to this method, we can use RSSs to forward the message in the gap area, and the message broadcast time can be calculated by Equation (2), the same equation used in Case A, by taking into account intermediate vehicles and RSSs as the required hops *s*.

(6) $$\frac{\sum_{k=1}^{K}\sum_{j=1}^{J}\frac{{\text{dist}}_{J_{j},K_{k}}-R_{c}}{\upsilon_{K_{k}}-\upsilon_{J_{j}}}}{K*J}$$

**Figure 7.**
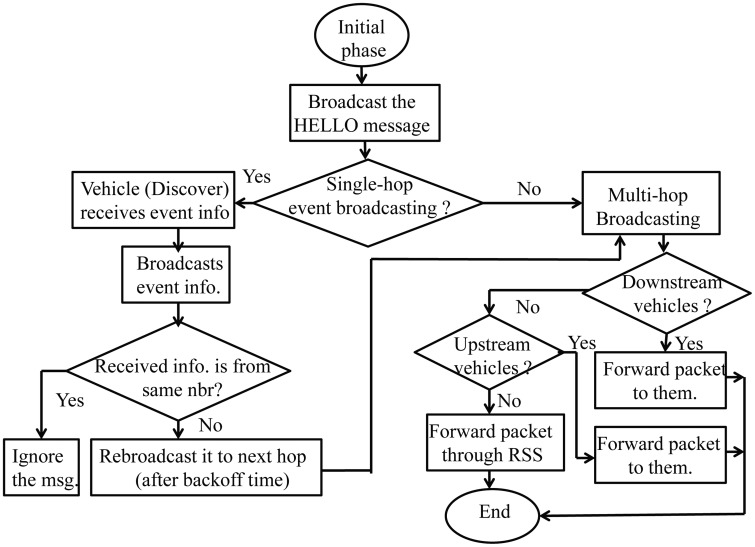
Flow chart for data transmission protocol in SVANET.

The complete flow of our proposed data transmission protocol is shown through the state transition diagram, as given in Figure 7.

### 4.4. Dynamic Channel Interval Adjustment (DCA)

According to IEEE 1609.4, the channel time is divided into sync intervals with a fixed duration of 100 ms for each interval, which consists of two equal parts: CCH and SCH. We use the number of neighbors and events to calculate the value of channel interval adjustment (*V_DCA_*) dynamically and use this value to setup the next new CCH interval. Since the number of events is very unpredictable, each CCH interval can be changed more frequently and dynamically. Hence, we calculate the value of the CCH interval by using the average number of *k* times of messages transmitted within each CCH interval. The packets include safety messages (*S*), HELLO messages (*H*) and WSA. In the next *k* CCH intervals, each vehicle will adjust the CCH interval based on the number of neighbors and events. Thus, Equation (7) is used to calculate the value of dynamic channel interval adjustment (DCA).

(7) $$V_{\text{DCA}}=\frac{\sum_{i=1}^{k}S_{i}+H_{i}+{\text{WSA}}_{i}}{k}$$

If vehicles have a larger DCA value, this means that they need more time to complete their transmissions during CCH. In other words, we need to give them more of a chance to announce their DCA value. As a result, the backoff time to broadcast the larger DCA value should be smaller. Vehicles having a larger DCA value will broadcast the DCA packet to their one-hop neighbors and User vehicles will drop their own DCA packet upon receiving the packet from the Providers, if it has a larger value. User vehicles will adjust their CCH interval based on the received DCA value in order to match the actual need without wasting bandwidth. In our protocol, the channel interval adjustment for two different packet types and four different traffic types are considered. The packet types are of the safety or non-safety type. The traffic types include a few safety messages and a large number of non-safety messages, a large number of safety messages and fewer non-safety messages, few safety messages and non-safety messages and a large number of safety messages and non-safety packets. We propose here to adjust the CCH duration for the first two types of traffic, as there is no need to adjust the CCH duration for the last two types. In the third case, a node should consider the channel duration, as given in the IEEE 1609.4 standard, and no channel interval adjustment is necessary, because the channel duration is enough for the low traffic load. In the last case, if a user receives a large number of safety and non-safety packets, it is suggested that multiple channels be used to handle the large volume of safety and non-safety messages, which can be achieved by using efficient multi-channel allocation algorithms.

It is also possible that a user may receive a large number of safety packets and few non-safety packets or few safety packets and a large number of non-safety packets. In this situation, the SCH interval should be changed to accommodate the required number of safety and non-safety messages. For example, as per the existing standard, the CCH and SCH duration is 50 ms each. However, based on our protocol, the duration of the CCH and SCH interval could be adjusted to 20 and 80 ms, respectively, if a few safety packets and a large number of non-safety packets are received. Although, according to our prediction, the CCH interval is too long, and the interval can be terminated by sensing CCH idle for a time period. If the control channel idle time is larger than the maximum waiting time of WSA, which implies that the neighbors do not want to use the control channel, the protocol stops the CCH interval and allocates the CCH remaining slots to SCH.

## 5. Leader Selection in SVANET

Normally, the data generated in a vehicular *ad hoc* network could be of the safety or non-safety types. As mentioned in Section 3, we classify the non-safety types of data into three different categories with different priorities, as given in Table 1. When people drive through the highway for many hours, the fellow passengers may often need different types of data, and we are of the mind that different types of data can be broadcast and shared in some specific service channels instead of switching to all channels. In the multi-channel, multi-user environment, though a user may get the channel, it may not get the required data of his or her choice. Moreover, a user has to scan all six service channels to get the data of one's choice. In order to minimize the switching delay and to improve the degree of rendezvous, we would like to propose here a data type-based channel switching mechanism, in which a leader has to broadcast the data, and other nodes within communication range of the leader can share it. Hence, in the following subsection, we are going to propose the leader selection algorithm.

### 5.1. Leader Selection Phase

It is assumed that different vehicles driving through the highway have different types of data to share with each other. The nodes (vehicles) having data to share with others are termed Providers. The nodes who get data from the Providers are termed as Receivers. Nodes that are neither Providers nor Receivers are termed as the No Data nodes. The Providers may have different types of data, and for each data type, we are going to select a Leader. Thus, four kinds of nodes, such as Leader, Provider, Receiver and No Data nodes, are considered in our work. For example, within the communication range of 250 m, suppose that there are 50 nodes, out of which 10 nodes have Type I data (multimedia data), eight nodes have Type II data (audio and web advertisement) and five nodes have Type III non-safety, non-real-time data, such as text messages. Here, we would like to design an algorithm to select a leader node for each data type, which will provide data to other users based on their interest. This implies that one node out of 10 nodes will be a leader to broadcast the Type I data, one node out of eight nodes will be selected as a leader to broadcast the Type II data and, similarly, one node from the five nodes will be selected to broadcast the Type III data. The rest of the nodes may get access to different types of data of their interest or may not use any service channel, which are termed the No Data nodes. However, if an event occurs, a warning message is triggered at the SOBU of each vehicle, and all nodes have to listen to the control channel for a certain duration to know about the event information. The situation is similar to the announcement of safety messages and the broadcasting of audio and videos inside a flying airplane through different channels. Here, the difference is that the cluster head has to rebroadcast the safety message to the next hop cluster head until the message is forwarded to the SRSU.

**Figure 8.**
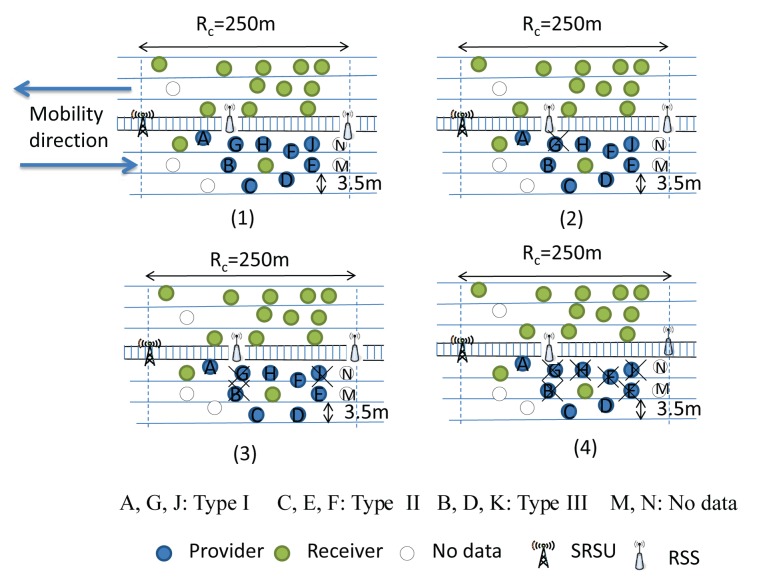
Selection of Leaders with different data types.

It is to be noted that the leader selection phase is executed among the vehicles within the same communication range. At the beginning of the selection phase, each node will go for a random backoff delay. The node having the least value for the random backoff delay has to change its role to Provider if it has any type of data to share with others. Then, it broadcasts a leader selection message with its data type information to let its one-hop neighbors know about its data type. Other providers who have the same type of data have to terminate their backoff delay and change their role to a Receiver and cannot be a member of the Leader selection procedure. However, Providers having different types of data to share with others will ignore that leader selection message. They continue to decrease their backoff timer and broadcast the leader selection message with their data type. The node that broadcasts the first leader selection message considers itself as the Leader after a certain predefined time interval. This procedure is continued until the leaders for those three types of data are selected. As shown in Figure 8(1), we consider that all vehicles are within the communication range and are moving along the same direction.

**Figure 9.**
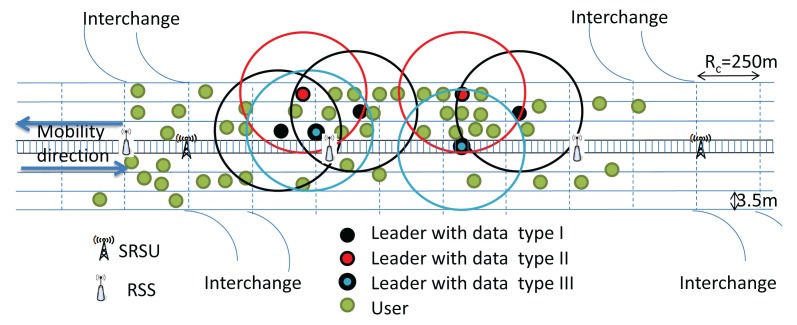
Leader selection without event.

Let Nodes *A* and *G* have Type I data. Nodes *C*, *E*, *F* and *H* have Type II data, and Nodes *B*, *D* and *K* have Type III data, whereas *M* and *N* are No Data nodes. Each provider has a different backoff time. Let the backoff time of Provider A(*P_A_*) be three units, and it has Type I data to share with others, as shown in Figure 8(2). As soon as *P_A_*'s backoff time is decreased from three to zero, *P_A_* will set itself as the Leader and broadcast the leader selection message. Let *P_G_* be the one-hop neighbor of *P_A_* and have the same Type I data to share with others. Upon receiving the leader selection message from *P_A_*, it has to terminate its backoff procedure and change its role to Receiver. Let *P_D_* be another one-hop neighbor of *P_A_*, who has Type III data to share with others, and its backoff duration is five units. After three units, though *P_D_* receives the leader selection message from *P_A_*, it ignores the message, as it has Type III data to share. It continues to decrease its backoff counter from eight to five to zero and follows the same procedure as *P_A_*. Since, Nodes *B* and *K* have the same Type III data, they have to terminate their backoff procedure and change their role to Receiver, as shown in Figure 8(3). Similarly, let backoff time of node *C* be eight units, and both Nodes *D* and *C* broadcast the leader selection message. However, since both nodes have different types of data to broadcast, they ignore each other's message. Since, Nodes *E*, *F* and *H* have Type II data and have a longer backoff duration, they have to terminate their backoff procedure and abort to be a leader, as shown in Figure 8(4). Finally, three identical nodes are selected as Leaders, as shown in Figure 9.

In order to decide the backoff time of a node, we consider the four different parameters of a node, including its data types (datatypes), number of receivers as neighbors (#receiver), number of no data nodes as neighbors (#nodata) and the average current speed of that vehicle. Calculation of the backoff time of a vehicle is given in Equations (8) and (9).

(8) $$\begin{array}{c} CL=\alpha_{CL}(\#\text{datatypes})*\beta_{CL}(\#\text{receiver})\\ {}*\gamma_{CL}(\#\text{nodata})*\mu_{CL}\left(1-\frac{\left|\text{speed}-\text{speed}d_{\text{avg}}\right|}{{\text{speed}}_{\text{avg}}}\right) \end{array}$$

(9) $$\begin{array}{c} \alpha_{CL}+\beta_{CL}+\gamma_{CL}+\mu_{CL}=1,\\ \alpha_{CL},\beta_{CL},\gamma_{CL},\mu_{CL}\in [0,1]. \end{array}$$

where *α_CL_*, *β_CL_*, *γ_CL_*, *μ_CL_* are the weights for the category of data type, number of receivers and number of no data nodes as neighbors, as well as the average speed of the vehicle, respectively.



**Algorithm 1** Leader competition algorithm.
1:computes *CL* value;2:listens to CCH;3:**if** CCH idle **then**4: **if**
*idletime* ≥ *AIFS*[*AC*_*VO*] **then**5:  broadcasts safety message;6: **else if**
*idletime* ≥ *AIFS*[*AC*_*VI*] **then**7:  broadcasts HELLO message;8: **else if**
*idletime* ≥ *AIFS*[*AC*_*BE*] **then**9:  broadcasts CL message;10: **else if**
*idletime* ≥ *AIFS*[*AC*_*BE*] **then**11:  broadcasts control message;12: **end if**13:**end if**14:**if** receives HELLO message **then**15: updates neighbor table;16: recompute *speed_avg_*,*CL*;17:**else if** receives Leader message **then**18: **if**
*event* = *True*
**then**19:  stop broadcasting own Leader message;20: **else if**
*event* = *False*
**then**21:  **if**
*type* = *type_self_*
**then**22:   drops its own Leader message;23:  **else if**
*type*! = *type_self_*
**then**24:   broadcasts own Leader message;25:  **end if**26: **end if**27:**end if**


After the leader selection phase is over, each Receiver has to tune its radio to the respective service channels, as given in Table 1. During transmission of the data in a different service channel, if any event occurs, the vehicle that detects the event has to send a warning message to all leaders, which is informs all Receivers to tune to the control channel (CCH). All leaders have to suspend the data transmission temporarily and listen to the control channel, too. The event-related safety message is then broadcast to the no data nodes for forwarding to the next hop vehicles and, ultimately, to the SRSU. The no data nodes check that the data relate to the event or not. If those data are related to an event, the no data nodes have to switch to any service channel to receive all data. After transmission of the data by a leader is completed, a leader has to inform its receivers with a data completion message. Then, the receivers have to compete again to be the next leader or a receiver. The leader competition algorithm is shown in Algorithm 1. The data transmission procedure by the Leader and receiving data by the Receiver is given in Algorithms 2 and 3, respectively.



**Algorithm 2** Data transmission procedure by the Leader.
1:computes position;2:Broadcasts Leader message;3:**if**
*event* = *True*
**then**4: collects data from discoverers;5: switches to SCH1;6: Transmission data;7: Broadcasts switch message;8: switches to SCH3;9: Transmission data;10: Broadcast finish message;11: switches to CCH;12: Broadcast finish message;13:**else if**
*event* = *False*
**then**14: switches to SCH1;15: Transmission data;16: Broadcast finish message;17: switches to CCH;18: Broadcast finish message;19:**end if**


**Algorithm 3** Receiving procedure of data by the Receivers.
1:receive Leader message;2:switch to SCH1;3:receive switch message;4:**if** want another type data **then**5: switch to SCH3;6: receive finish message;7: switch to CCH;8:**else**9: switch to CCH;10:**end if**


### 5.2. Next Leader Selection Phase

Once the data transmission by a Leader is completed, the next leader selection phase is started. The backoff time for selecting the next leader is calculated as given in Equations (10) and (11). We consider the type of forwarding data, projection distance, position, number of neighbors and average speed of the vehicles as parameters to select the next leader.

(10) $$\begin{array}{c} nL=\alpha_{nL}\left(\frac{\text{Project distance from self to Leader}}{R_{c}}\right)*\\ \beta_{nL}(\text{position})*\gamma_{nL}(\text{number of neighbor})*\delta_{nL}\left(\text{speed}\right) \end{array}$$

(11) $$\begin{array}{c} \alpha_{nL}+\beta_{nL}+\gamma_{nL}+\delta_{nL}=1,\\ \alpha_{nL},\beta_{nL},\gamma_{nL},\delta_{nL}\in [0,1]. \end{array}$$

where *α_nL_*, *β_nL_*, *γ_nL_*, *δ_nL_* are weights for projection distance, position, number of neighbors and average speed of the vehicle, respectively.

In our protocol, the vehicle that is farthest from the position of the current leader is selected as the next leader. However, if a vehicle is farthest from the current leader and is already within communication range of the SRSU, then that vehicle is not considered as the next leader, as it can move away from the communication range of the SRSU after a certain interval of time. Besides, the next leader selection also depends on the distance between the no data vehicle and the leader and a vehicle's mobility direction. If the no data vehicle moves along the same direction of the existing leader, then the no data node with the farthest distance will be chosen as the leader. However, the nearest node to the existing leader is selected as the new leader if its mobility direction is opposite of the mobility direction of the existing leader.



**Algorithm 4** Next Leader competition for the No Data vehicles.
1:receive Leader message;2:**if**
*event* = *True*
**then**3: switch to SCH1;4: Receive data;5: receive switch message;6: switch to SCH3;7: receive data;8: receive finish message;9: switch to CCH;10: compute *nL* value;11: broadcast nL message;12: receive nL message;13: **if**
*nL* < *nL_self_*
**then**14:  broadcast Leader message;15: **else if**
*nL* > *nL_self_*
**then**16:  silence;17: **end if**18:**else if**
*event* = *False*
**then**19: stay in CCH;20:**end if**


**Algorithm 5** Next Leader competition for the Providers.
1:receive Finish message;2:**if**
*event* = *True*
**then**3: keep silence and stay in CCH;4: Receive data;5: receive switch message;6: switch to SCH3;7: receive data;8: receive finish message;9: compute *nL* value;10: broadcast nL message;11: receive nL message;12: **if**
*nL* < *nL_self_*
**then**13:  broadcast Leader message;14: **else if**
*nL* > *nL_self_* then15:  silence;16: **end if**17:**else if**
*event* = *False*
**then**18: **if**
*type*! = *type_self_*
**then**19:  keep silence and stay in CCH;20: **else if**
*type* = *type_self_*
**then**21:  compute *nL* value;22:  broadcast own Leader message;23: **end if**24:**end if**


**Figure 10.**
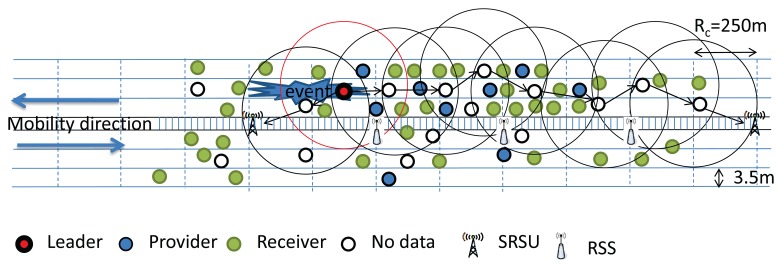
Selection of next Leader when vehicles move along the same direction.

**Figure 11.**
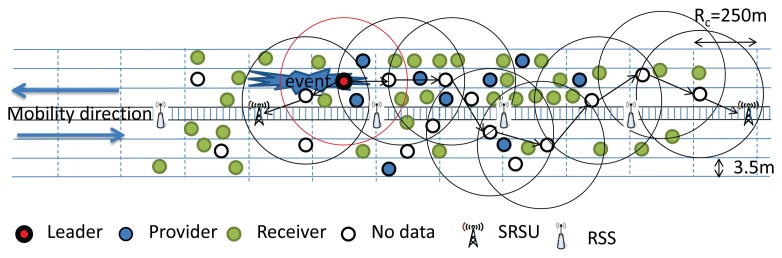
Selection of the next Leader when vehicles move along the opposite direction.

**Figure 12.**
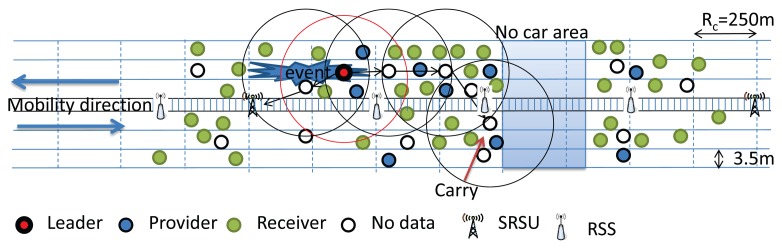
Selection of the next Leader when the network is partitioned.

If there are two no data vehicles within the same communication range, they may interfere with each other. In this case, we choose a node as the next leader based on a node's backoff time, as given in the Equation (10). If two no data vehicles are not within the same communication range, they will not interfere with each other, and therefore, both of the no data vehicles could be the next leader. The overlapping area between the two new leaders may have interference if they use the same channel. However, the vehicles in this area must have received data from the previous leader, and therefore, we think the interference is not important here. According to the location of the vehicles along both sides of the highway, we can use different paths to transmit data to the SRSU. We can get a path to transmit data through the vehicles moving along the same or opposite direction, as shown in Figures 10 and 11, respectively. If no vehicle is within communication range of others and the network is partitioned, then the existing leader has to carry the data until it comes within the communication range of some other vehicle, as shown in Figure 12. However, the event information can be transmitted through the RSS to the SRSU, though the data cannot be transmitted. If the selected next leader is not meant for transmitting the event data, then the new leader is selected based on the requirement of the data. The providers who have the requested types of data will compete to be the next leader. We calculate the new *nL* value without any event, as given in Equation (12). The algorithms of the competition of the leader for the No Data vehicles and Providers are shown in Algorithms 4 and 5, respectively.

(12) $$\begin{array}{c} \text{nnL}=\alpha_{nL}\left(\frac{1}{\text{Project distance from self to Leader}}\right)*\\ \beta_{nL}(\text{position})*\gamma_{nL}(\text{number of neighbor})*\delta_{nL}\left(\text{speed}\right) \end{array}$$

## 6. Performance Evaluation

The performance evaluation of our proposed hybrid vehicular *ad hoc* networks with wireless sensors is conducted using the NS2 simulator with MOVE [32], which is a realistic mobility model and can be immediately used by NS2. The simulation environment and corresponding results are described as follows.

### 6.1. Simulation Setup

In our performance evaluation, a highway scenario with six and eight lanes on both sides of the road is considered. The average speed of each vehicle is 100 km/h, and the safe distance between any two vehicles running along the same lane is 30 m. The distance between any two SRSU is considered to be 5 km, and the communication range of each vehicle and the SRSU are 250 m. In between any two SRSUs, RSSs are positioned at a distance of 1 km from each other to collect the data from the wireless sensors of vehicles. Thus, in between two SRSUs, there are four RSSs. Communications between sensors and RSSs use the IEEE 802.15.4 protocol, while communications between the SOBU and SRSU use the IEEE 802.11p protocol. The number of vehicles in the six-lane highway is taken to be 100 through 600 and in the eight-lane highway 100 through 800 within the communication range of 250 m.

In our simulation, it is assumed that half the number of vehicles in a cluster has no data to send or receive. Hence, these no data vehicles are used for the leader selection procedure to transmit event information from one SRSU to another. The packet interval rate and packet size are set to be 0.01 s and 512 bytes, respectively. The safety packets are generated randomly, and the CCH interval is considered to be within 1 ms through 100 ms. The distance between any two SRSUs is divided into several clusters based on the communication range of 250 m. In order to evaluate the performance of our protocols, we analyzed using Equations (13)–(16). Different CCH intervals *a* are generated randomly using Equation (13). This generated random value *a_i_* for *i* varying from one through *k* is considered, and the average value (*AVG*) is calculated for *k* = 10, as given in Equation (14). Taking the previous and current random value *a_i_*_−1_ and *a_i_*, respectively, a new value *x* is calculated based on the standard and our protocol, as given in Equation (15). Then, a new function is derived using the value of *x* as the input to evaluate if the channel is busy (*B*) or idle (*I*), as given in Equation (16).

(13) $$\{\begin{array}{ll} a=\text{random}\left(0,46\right), & \text{CCH}\;\text{interval}<50ms\\ a=\text{random}\left(47,92\right), & 50ms\geq \text{CCH}\;\text{interval}\leq 100ms\\ a=\text{random}\left(0,92\right), & 1ms\geq \text{CCH}\;\text{interval}\leq 100ms \end{array}$$

(14) $$\text{AVG}=\frac{\overset{k}{\underset{i=1}{\text{∑}}}a_{i}}{k}$$

(15) $$\{\begin{array}{ll} x_{i}=a_{i}-46, & \text{Standard}\\ x_{i}=a_{i}-a_{i-1}, & \text{each time change}\\ x_{i}=a_{i}-\text{AVG}, & \text{ours} \end{array}$$

(16) $$f\left(x\right)=\{\begin{array}{ll} B+x, & \text{if}\;x>0\\ I+|x|, & \text{if}\;x<0 \end{array}$$

### 6.2. Simulation Results

**Figure 13.**
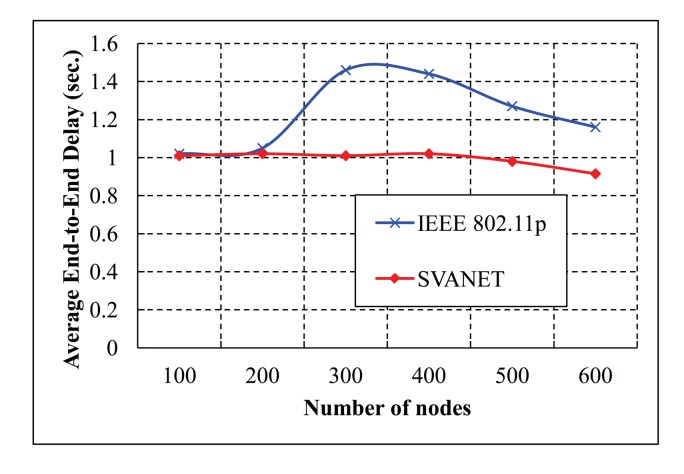
The number of packets dropped at the SRSU, when the CCH interval is between 50 ms and 100 ms.

**Figure 14.**
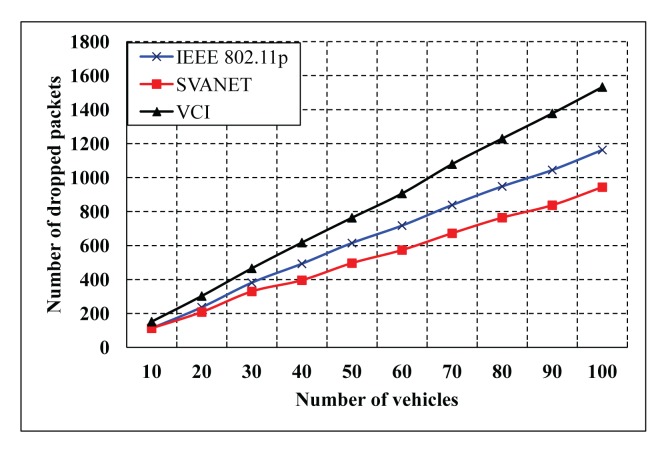
The number of packets dropped at the SRSU when the CCH interval is between 1 ms and 100 ms.

In our evaluation, we compare our protocol (SVANET) with IEEE 802.11p in terms of the packet delivery ratio, average end-to-end delay and first packet received time at the SRSU. Besides, the number of selected leaders is also simulated for the six- and eight-lane highway. Our protocol is also compared with VCI [16] in terms of the number of forwarded packets in the CCH interval. In the simulation results, the number of dropped packets that is defined as the number of safety and non-safety packets could not arrive at the SRSU, and the number of nodes is defined as the sum of vehicles, sensors and RSSs. The simulation results are shown in Figures 13 and 14, where the CCH interval is between 50 ms and 100 ms and between 1 ms and 100 ms, respectively. In Figure 13, we find that the number of dropped packets in the standard is greater than the VCI and SVANET. As the duration of the CCH interval in the standard is fixed, it cannot complete all of the packet transmissions. In this case, the number of dropped packets of the VCI is less than our protocol when the number of nodes is less than 40. However, SVANET is better than VCI in terms of the total number of dropped packets. In Figure 14, the number of dropped packets is also simulated for the random duration of the CCH interval. It is observed that our protocol outperforms the standard and VCI, as we consider dynamic change in the sync interval for the durations of CCH and SCH.

**Figure 15.**
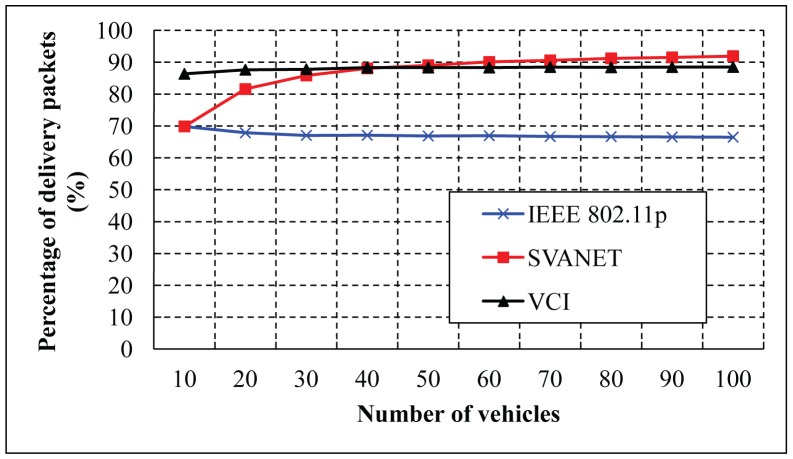
Percentage of safety packets delivered to the SRSU when the CCH interval is >50 ms.

**Figure 16.**
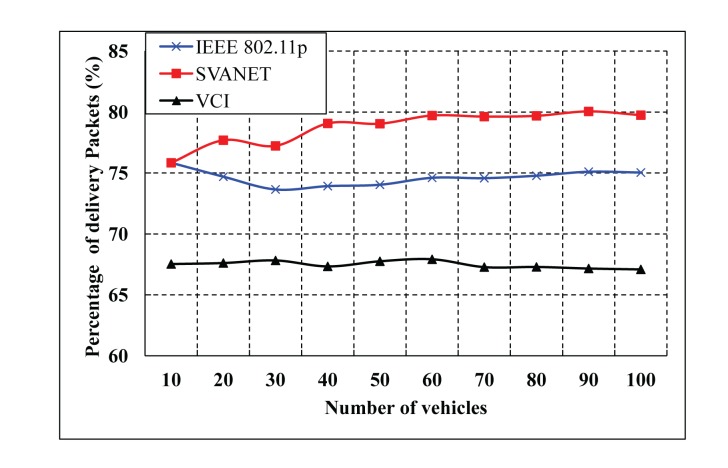
The percentage of safety packets delivered to the SRSU when the CCH interval varies from 1 to 100 ms.

The percentage of delivered packets for different numbers of vehicles is shown in Figures 15 and 16. Here, the delivered packets are considered to be the safety type, which are simulated for the CCH interval >50 ms and between 1 ms and 100 ms. In these simulations, we consider only the number of vehicles to deliver the packets to the SRSU. In both cases, SVANET outperforms VCI and the standard, considering a fixed duration of CCH and SCH. In our protocol, the duration is dynamic, and the channel can be adjusted dynamically to transmit the packets.

**Figure 17.**
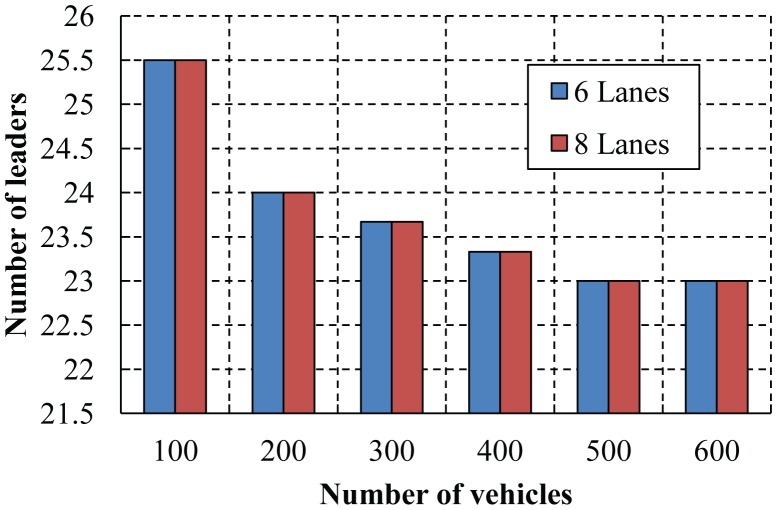
The number of selected leaders for different numbers of vehicles.

As shown in Figure 17, the number of leaders selected according to our algorithm for different numbers of vehicles is simulated. In the case of a low density of vehicles, more leaders are selected as the connectivity is difficult to maintain among vehicles. The number of leaders is the same irrespective of the number of lanes, as the density of the nodes plays an important role to select the leaders.

**Figure 18.**
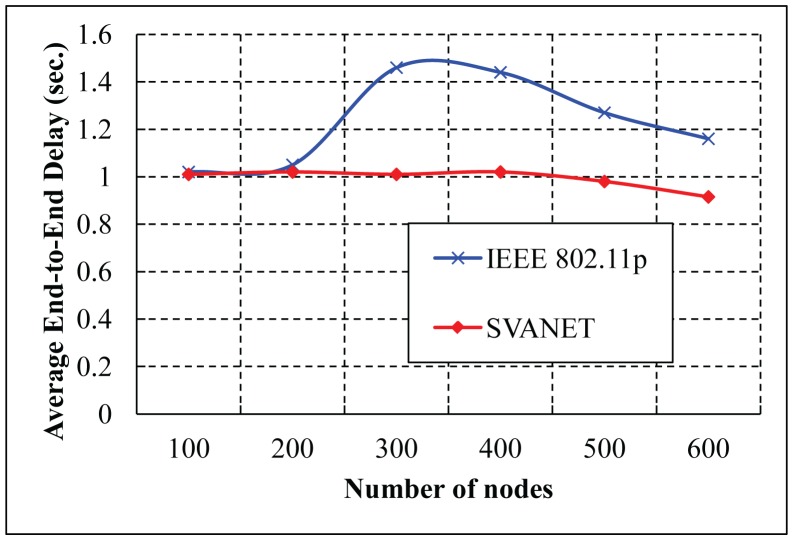
Average end-to-end delays in the highway with six lanes.

**Figure 19.**
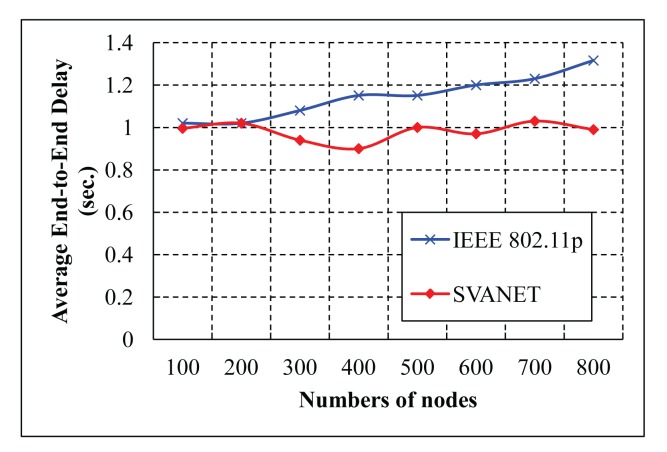
Average end-to-end delays in the highway with eight lanes.

The average end-to-end delay of transmitting packets from the vehicles to the SRSU is simulated for the highway with six and eight lanes, as shown in Figures 18 and 19, respectively. From the simulation results, we observe that the average end-to-end delay in SVANET is smaller than the standard, as the data is transmitted through the leaders, as well as the RSSs. In SVANET, the average end-to-end delays are almost the same, no matter which number of vehicles is simulated. The reason is that, since our packet transmission is achieved with the help of leader nodes, the density of the vehicles does not affect the delay, except the case when the network is partitioned.

**Figure 20.**
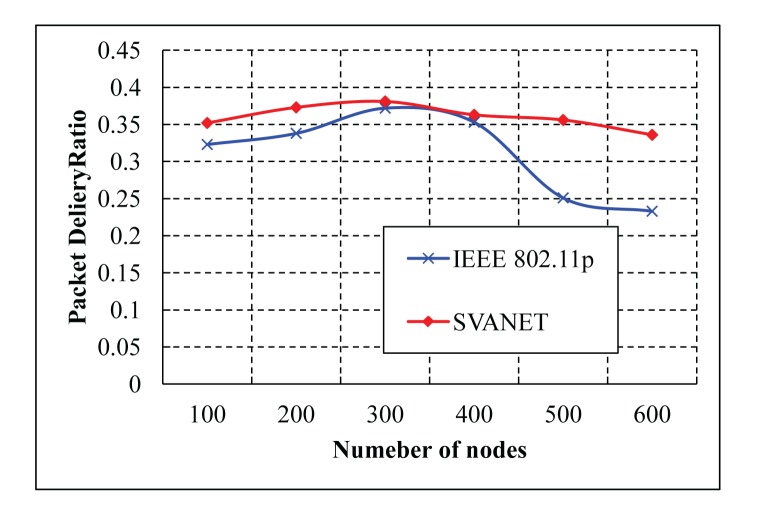
Average packet delivery ratios in the highway with six lanes.

**Figure 21.**
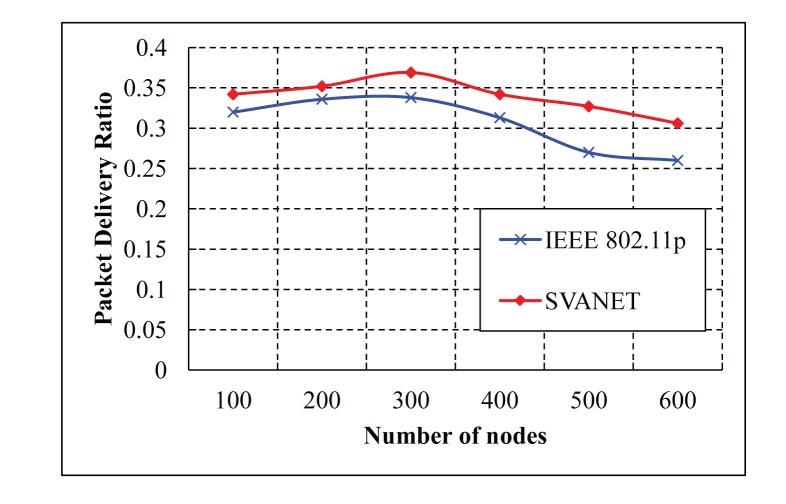
Average packet delivery ratios in the highway with eight lanes.

**Figure 22.**
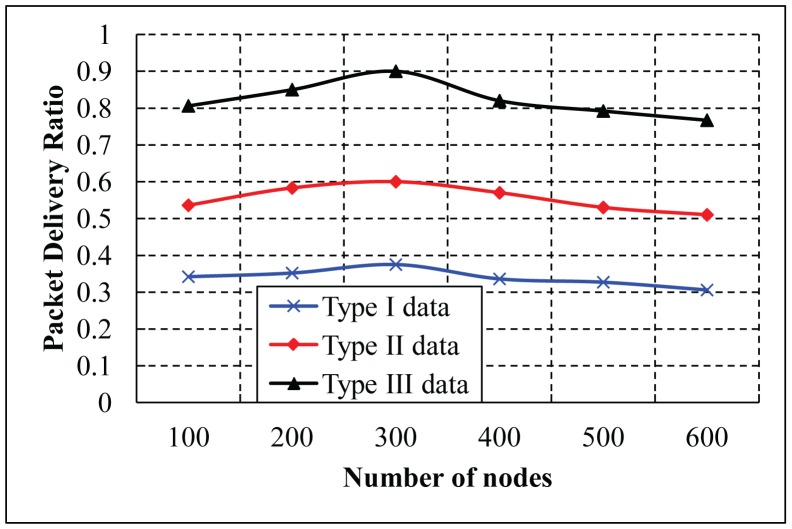
Average packet delivery ratios for different data types with eight lanes.

The average packet delivery ratio with different numbers of nodes is simulated in the highway scenario with six and eight lanes, as shown in Figures 20 and 21, respectively. As shown in the two simulation results, it is observed that SVANET outperforms the standard in terms of the packet delivery ratio. The reason is that, during the packet forwarding process, some vehicles that carry the packets might exit through the interchanges, such that the packet delivery will be interrupted. However, in SVANET, though vehicles also can exit through the interchanges, sensors and road side sinks can help to forward the packets from one hop to another. Even if the network is partitioned due to the absence of vehicles, road side sinks and sensors can help to forward the packets to the SRSU. Though we have simulated the packet delivery ratio using wireless sensors, road side sinks and vehicles for the highway with six and eight lanes, we do not find any visible change in the delivery of the packets. This happens due to the use of leaders and RSS to transmit the packets to the SRSU. In order to analyze the effect of data types on the packet delivery ratio, we simulated SVANET for different sizes of packets, 512, 256, 128 bytes, as data Types I, II and III, respectively. The average packet delivery ratio for different data types with a different number of vehicles is simulated in the highway scenario with eight lanes, as shown in Figure 22. From the simulation result, it is found that the packet delivery ratio of Type I data is higher than others. This is due to the nature of the data, because it is text messages only.

## 7. Conclusions

In this paper, a data transmission protocol for forwarding different types of data is proposed. A hybrid VANET architecture that is different from the existing research is proposed to form a smart vehicular *ad hoc* network with a limited number of sensors and road side sinks. Towards addressing the existing problem of data transmission and sharing, we propose the idea of integrating the VANET with inexpensive wireless sensors. Sensor nodes are fitted in vehicles and road side sinks deployed along the roadside to sense any kind of road conditions and to buffer and deliver messages to other vehicles, regardless of the density or connectivity of the VANET. In this paper, we investigate these challenges and propose schemes for effective and efficient vehicle-to-sensor and sensor-to-sensor interactions. A multi-channel communication mechanism between the sensors and vehicles is proposed in our SVANET, in which both IEEE 802.15.4 and IEEE 802.11p MAC are used. Besides, based on the IEEE 1609.4 standard, a dynamic channel adjustment protocol is designed, which can adjust the interval between CCH and SCH to transmit messages efficiently. In order to improve the data delivery ratio and data transmission reliability, a leader selection algorithm among vehicles is proposed, so that different types of data, such as video, audio and text, can be transmitted to passengers with other vehicles based on their interest. Based on our method, leaders can transmit the data to SRSUs and vehicles through SRSUs and RSSs. Since, drivers can get the event information, such as icy road conditions, accident locations and traffic congestion in advance, they can find alternate routes to exit through nearby interchanges. We developed algorithms to adjust the CCH and SCH intervals dynamically and have compared the performance of our protocol with the IEEE 802.11p standard. The proposed SVANET can be economically feasible as there is a small number of road side units and sensors used to improve the average data delivery ratio irrespective of the number of vehicles in each lane on the road.

## Figures and Tables

**Table 1.** Priority based data classification with channels. CCH, control channel; SCH, service channel.

**Types of Data**	**Category of Data**	**Priority**	**Channel**
Safety and Control message	Urgent	1	CCH
Video and graphics data	I	2	SCH1 and SCH2
Audio, images and advertisements	II	3	SCH3 and SCH4
Text messages and emails	III	4	SCH5 and SCH6
